# Advances in vibration therapy for the treatment of osteoporosis

**DOI:** 10.3389/fendo.2025.1611677

**Published:** 2025-08-18

**Authors:** Xueyan Lu, Haoyang Duan

**Affiliations:** ^1^ Department of Rehabilitation Medicine, Tonghua City People’s Hospital, Tonghua, China; ^2^ Department of Rehabilitation Medicine, First Hospital of jilin University, Changchun, China

**Keywords:** vibration therapy, osteoporosis, bone density, osteoblasts, osteoclasts, mechanical stress

## Abstract

Osteoporosis is a systemic skeletal disease characterized by reduced bone density and degeneration of bone microstructure. It is prevalent among postmenopausal women and elderly individuals. Current treatments face challenges such as drug side effects, low adherence, and comorbidities. Vibration therapy, as a non-invasive physical treatment, regulates bone metabolism through mechanical stress stimulation and is emerging as an important complementary strategy in the comprehensive management of osteoporosis. This article systematically reviews the mechanisms of action and clinical efficacy of vibration therapy. Studies indicate that vibration therapy activates osteoblast differentiation pathways (e.g., the Wnt/β-catenin signaling pathway) through low-frequency mechanical vibrations, upregulates osteogenic markers (e.g., Runx2, BMP-2, OPG), and inhibits osteoclast activity, reducing the RANKL/OPG ratio to bidirectionally regulate bone metabolic balance. Further mechanistic studies on muscle dynamics show that vibration stimulation enhances muscle contractile force, promoting bone formation through mechanical loading. Clinical trials demonstrate that vibration therapy has potential in improving lumbar and hip bone density, enhancing bone biomechanical properties, and reducing fracture risk, particularly when combined with drugs such as bisphosphonates or teriparatide, showing synergistic effects. However, variability in therapeutic outcomes (e.g., insignificant improvement in trabecular structure) may be related to differences in vibration parameters (frequency, amplitude, acceleration), device types, and individual responses. The current advantages of vibration therapy lie in its ease of use, high safety, and good adherence, but its clinical application still lacks standardized parameter guidelines. Future research should establish individualized treatment protocols and a biological equivalent dose system through large-scale randomized controlled trials to promote the standardized development of this therapy.

## 1 Introduction

Osteoporosis is a systemic bone disease. Its main pathological features are significantly lower bone density, progressive degeneration of bone microstructure, and reduced bone mechanical strength. Patients can fracture under low - energy trauma or normal activity - related loads (1). It’s a systemic bone tissue degenerative lesion due to bone metabolic imbalance. Its pathogenesis involves reduced osteoblast activity and increased osteoclast activity, leading to bone remodeling imbalance (2, 3).

Osteoporosis is divided into primary and secondary types. Primary osteoporosis includes type I (postmenopausal), caused by estrogen deficiency; type II (senile), linked to age - related bone metabolic imbalance; and idiopathic (more common in the young and middle - aged), with an unclear cause. Secondary osteoporosis refers to bone metabolic disorders caused by specific pathological or exogenous factors, such as endocrine disorders, chronic kidney disease, long - term glucocorticoid or antiepileptic drug use, which trigger abnormal bone loss (4).

Epidemiological studies show that osteoporosis mainly occurs in postmenopausal women and the elderly. In the global population aged 15–105 and above, the prevalence of osteoporosis is 18.3%, with a marked gender difference. The prevalence in women (23.1%) is roughly double that in men (11.7%) (5). In China, the prevalence of osteoporosis in people aged 50 and above is as high as 19.2%, with women’s prevalence reaching 32.1%, far higher than that of men (6.0%) (6). With the accelerating population aging, osteoporosis has become a major global public health challenge (7). Its disease burden is not only reflected in health issues but also causes huge social and economic losses. Take osteoporotic fractures as an example. The annual direct medical expenditure in the US and the UK is about $17.9 billion and £4 billion respectively, highlighting the urgency and economic value of preventing and controlling this disease (8).

At present, drug therapy is the main treatment for osteoporosis (9). Basic treatment focuses on calcium and vitamin D supplements to correct calcium and phosphorus metabolic imbalances, offering essential substrates for bone remodeling. Anti - osteoporosis drugs precisely regulate different pathological mechanisms. They include bisphosphonates (alendronate, zoledronic acid), RANKL inhibitors (denosumab), parathyroid hormone analogs (teriparatide), and vitamin D metabolites (calcitriol) and vitamin K2 derivatives (menaquinone-4) that regulate bone metabolism.Evidence - based medicine shows standardized drug intervention can improve bone metabolism indicators, relieve chronic bone pain, and reduce the risk of major osteoporotic fractures by 30% - 70%. Take bisphosphonates as an example. By inhibiting osteoclast activity, they balance bone remodeling. Long - term use can cut hip and spinal fracture risk by nearly 50% within three years and significantly improve vertebral trabecular microstructure and bone biomechanical properties (10).

Drug therapy for elderly osteoporosis patients faces many clinical challenges. First, multimorbidity (an average of 3.2 chronic diseases per elderly patient) limits drug options, with about 65% of patients having at least one contraindication to anti - osteoporosis drugs. Second, age - related physiological decline significantly increases the risk of adverse drug reactions (2.3 times higher than in younger patients). Common issues include bisphosphonate - related osteonecrosis of the jaw (incidence of 1.2 - 4.3%) and increased fracture risk from long - term proton pump inhibitor use. These factors together lead to significantly worse treatment adherence in the elderly, ultimately increasing their fracture risk compared to those with standardized treatment (11, 12).

In the comprehensive management of osteoporosis, rehabilitation therapy, as a non - invasive complement to pharmacological intervention (13), is gaining attention. Current physical therapy strategies include extracorporeal shock wave therapy, pulsed ultrasound, mechanical vibration training, and customized exercise prescriptions. They work by applying targeted mechanical stress to regulate the biological behavior of key bone metabolism cells - osteoblasts and osteoclasts. Mechanical signals can activate osteoblast differentiation pathways (e.g., Wnt/β - catenin signaling cascades) and inhibit osteoclast precursor fusion, promoting bone matrix mineralization and suppressing bone resorption (14).

Vibration therapy, with its systemic impact and cost - effectiveness, is emerging as an important supplement to local mechanical wave therapies like extracorporeal shock waves and ultrasound. Unlike localized physical interventions, it uses low - frequency mechanical vibrations (usually <50Hz) to provide overall skeletal system stimulation. Its cost - effectiveness is accelerating its application in clinical rehabilitation, especially in improving bone density and balance function.

## 2 What is vibration therapy

As an important branch of physical medicine, the historical origins of vibration therapy can be traced back to ancient Greece, where physicians used simple mechanical devices for localized vibration treatment to improve bodily functions. Despite centuries of clinical practice, this therapy long remained a supplementary treatment. With advancements in modern biomechanics and rehabilitation medicine, systematic vibration therapy—characterized by standardized operational protocols, strong patient tolerance, and quantifiable therapeutic outcomes—has gradually evolved from traditional empirical medicine to evidence-based modern rehabilitation technology. Currently, this technique has expanded beyond orthopedics and established specialized applications in neurological rehabilitation, demonstrating unique efficacy in areas such as Parkinson’s disease-related motor disorders, cerebral palsy muscle tone regulation, and balance function restoration in multiple sclerosis (15, 16).

Vibration therapy is a physical treatment technology based on biomechanical principles, utilizing precision equipment to generate mechanical vibration waves with specific frequencies and amplitudes. It can be classified into two main types based on operational modes:① Focal Muscle Vibration (FMV): Targets specific muscle groups or joints with localized vibration sources, primarily stimulating the affected area and adjacent tissues.② Whole Body Vibration (WBV): Transmits vibrations through a platform to the entire skeletal muscle system via centrifugal conduction from the lower limbs, inducing systemic neuromuscular activation. Both modalities regulate stress adaptability in the musculoskeletal system through mechanical vibration signals, though their scopes and physiological effects differ significantly (17).The limitations of whole-body vibration therapy include restricted applicability: people with severe osteoporosis, cardiovascular disease, or pregnancy may face increased risk due to the systemic stress response. Furthermore, efficacy depends heavily on platform type (vertical vs. oscillatory) and parameter settings; improper operation can easily reduce effectiveness.

Vibration therapy can be further categorized multidimensionally based on core parameters:①Vibration vector direction: Includes vertical axial vibration (along the body’s longitudinal axis), transverse tangential vibration (parallel to the support plane), and lateral coronal vibration (along the left-right axis).②Energy conversion mechanisms: Divided into mechanical transmission vibration (generated by eccentic wheel devices), electrodynamic vibration (based on electromagnetic principles), and magnetostrictive vibration (utilizing magnetostrictive effects).③Vibration phase modes: Encompasses synchronous vibration (bilateral phases aligned) and alternating vibration (180° phase difference between sides). Different vibration patterns influence neuromuscular activation through distinct mechanical stimuli, with synchronous vibration providing symmetrical bilateral stimulation and alternating vibration inducing alternating muscle activation.

Vibration therapy achieves therapeutic goals by applying vibratory stimuli to local or systemic regions, triggering periodic or non-rhythmic physiological responses. Whole-body vibration therapy employs a specialized platform, with subjects adopting standing (bipedal/unipedal) or seated positions. The generated impact vibrations transmit through the lower limbs to bone tissue cells, enhancing musculoskeletal strength via bidirectional regulation of bone metabolism (promoting osteogenesis while inhibiting osteoclast activity) (18). Vertical-direction vibration produces maximal amplitude effects, promoting bone tissue proliferation and rhythmic muscle contractions through mechanical stimulation, now recognized as the mainstream modality. Typical parameters include: frequency 15–60 Hz, acceleration 0–15g (1g = 9.8 m/s²), and amplitude 1–15 mm. High-frequency (>20 Hz) combined with low-acceleration (<1g) parameters have been validated as optimal therapeutic doses (19).

## 3 Mechanisms of vibration therapy in treating osteoporosis

### 3.1 Bone conduction mechanism

Bone tissue contains a class of mechanosensitive cells that perceive mechanical stress changes through nuclear oscillations, triggering dynamic adjustments in bone mass and structure to adapt to mechanical loading demands (20). These mechanosensitive cells primarily include osteocytes, osteoblasts, and mesenchymal stem cells, which possess mechanosensing and response capabilities to regulate bone metabolism and remodeling (18).

**Figure 1:**
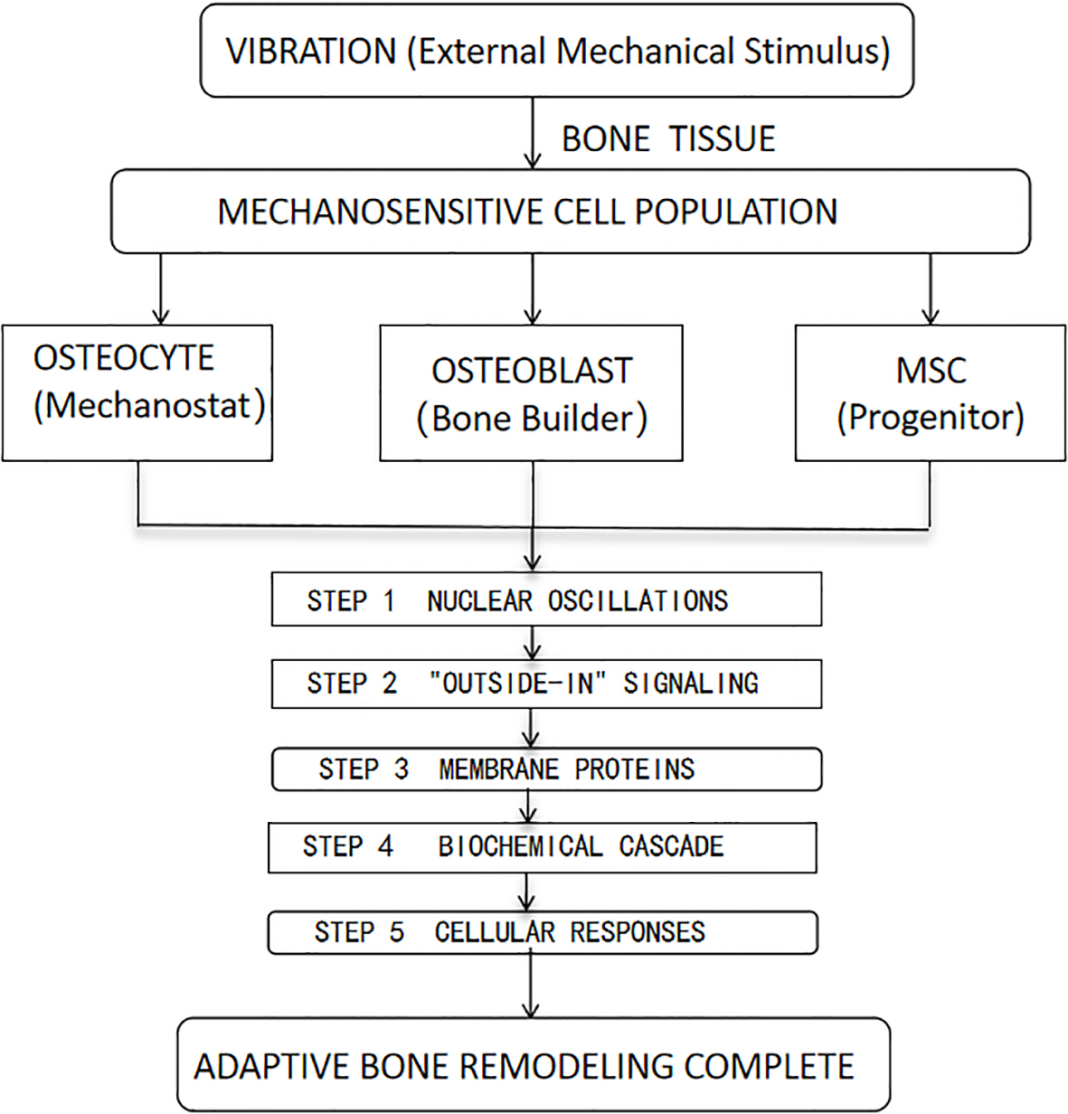
Diagram Key: MSC, Mesenchymal Stem Cell. HSC, Hematopoietic Stem Cell. The five numbered steps trace the mechanotransduction pathway from vibration sensing to tissue-level adaptation.

Vibration generates mechanical signals that promote metabolism in the bone and tendon systems. This “outside-in” signaling originates at the cell membrane, where mechanical stimuli are converted into biochemical signals via membrane proteins (21). Studies reveal that vibration-induced acceleration regulates bone remodeling responses, driving the differentiation of bone marrow mesenchymal stem cells and hematopoietic stem cells into specific lineages, thereby enabling adaptive remodeling of bone tissue (22) (**Figure 1**).

These studies emphasize the key role of mechanically sensitive cells in bone conduction. Mechanical signals from vibration can regulate bone metabolism and remodeling, supporting vibration therapy’s application in osteoporosis treatment. They also suggest future research directions, such as the specific effects of different vibration types on bone cells and their long - term impacts.

These studies emphasize the crucial role of mechanically sensitive cells in bone conduction. Mechanical signals generated through vibration can modulate bone metabolism and remodeling, providing a theoretical basis for the application of vibration therapy in the treatment of osteoporosis. They also indicate potential directions for future research, such as exploring the specific effects of different types of vibration on bone cells and their long-term impacts.

### 3.2 Bone metabolism mechanism

Vibration therapy effectively regulates bone metabolism by upregulating osteogenesis-related factors and enhancing osteoblast activity. Studies demonstrate that mechanical vibration significantly promotes osteoblast proliferation and mineralization, indicating its positive effects on bone formation processes (23). Further analysis reveals that vibration therapy markedly upregulates the expression of key osteogenic proteins, including alkaline phosphatase (ALP), osteocalcin (OCN), runt-related transcription factor 2 (Runx2), bone morphogenetic protein 2 (BMP-2), osteoblast-specific transcription factor Osterix, and osteoprotegerin (OPG)—all critical regulators of bone formation and osteoblast differentiation (24).

After applying vibration stimuli (45 Hz, 30 minutes/day for 5 consecutive days) to rat bone marrow mesenchymal stem cells (BMSCs), researchers observed significant upregulation of osteogenic markers (Runx2, collagen type I, osteocalcin) and increased expression of key components in the Wnt/β-catenin signaling pathway (osteoprotegerin, Wnt3a, β-catenin, and their mRNAs). These findings suggest that vibration therapy promotes osteogenic differentiation of BMSCs by activating the Wnt/β-catenin pathway, while significantly reducing osteoclast differentiation factor (RANKL) levels (P<0.05) and suppressing osteoclast activity, thereby achieving bidirectional regulation of bone metabolism (25).

Another study found that vibration therapy (30–35 Hz, 20 minutes/day, 5 sessions/week for 6 weeks) significantly decreased serum serotonin levels (P<0.001) in ovariectomized rats. Reduced serotonin—a bone metabolism regulator—correlated with improved femoral bone density (P<0.05) and biomechanical performance, alongside suppressed RANKL levels (P<0.001) and inhibited osteoclast differentiation. This reveals that vibration therapy exerts anti-osteoporotic effects by modulating the neurotransmitter-bone metabolism axis (26).

Additionally, vibration therapy significantly enhances Wnt signaling pathway-related protein expression while reducing the receptor activator of nuclear factor kappa-B ligand (RANKL)/osteoprotegerin ratio, thereby inhibiting osteoclast proliferation and promoting dynamic equilibrium between bone resorption and formation. This mechanism provides theoretical support for vibration therapy in bone metabolism regulation (27, 28).

These studies outline the mechanisms of vibration therapy in bone metabolism regulation, reveal its dual - regulatory effects on bone formation and resorption at molecular and cellular levels, provide a solid theoretical basis for its use in osteoporosis treatment, and highlight its potential clinical applications.

### 3.3 Muscle dynamics mechanism

The maintenance and increase of bone mass primarily depend on bone loading and mechanical stress, which originate from active muscle contractions. Muscle strength plays a decisive role in bone structure and mass, with changes in muscle strength typically preceding alterations in bone strength. Following natural menopause, muscle atrophy and weakened mechanical stimulation due to declining muscle strength contribute to osteoporosis onset. Data indicate that age-related osteoporosis in women is often accompanied by significant reductions in muscle strength (29).

Interventions targeting this issue show promising results. For instance, postmenopausal women with low bone mass who underwent resistance training twice weekly for 9 months exhibited significantly increased tibial distal bone density measured via peripheral quantitative CT. Furthermore, whole-body vibration therapy mimics the frequency range of muscle-generated impulses, inducing stretch reflexes and enhancing muscle contractions to generate mechanical stress on bone tissue, thereby promoting bone formation (30). Studies confirm that vibration therapy, similar to swimming and jumping exercises, increases bone mass, enhances bone strength, and stimulates osteoblast activity, effectively maintaining bone quality and preventing disuse osteoporosis (31).

These studies emphasize the significance of muscle strength for maintaining and increasing bone mass, and link the decline in muscle strength after natural menopause to osteoporosis development. Furthermore, the intervention measures highlighted, such as resistance training and whole - body vibration therapy, offer practical ways to prevent and improve osteoporosis, making them highly significant and valuable for clinical applications.

## 4 Therapeutic efficacy of vibration therapy in osteoporosis

Wei et al. demonstrated that both vibration therapy alone and herbal medicine independently prevented osteoporosis in ovariectomized rats, 50 ovariectomized rats were divided into five groups and after 12 weeks of treatment, levels of osteopontin (OPN), RANKL and bone turnover markers in serum were measured, and bone density (BMD), histomorphometry and bone strength were evaluated, their combination significantly enhanced bone mineral density (BMD), bone strength, and bone structure, thereby amplifying the therapeutic effects of vibration therapy (32). Camargos et al. explored the effects of high - frequency whole - body vibration (HF - WBV) and alendronate (ALN), used alone or together, on the bone mechanical properties of ovariectomy - induced osteoporotic rats. Thirty - four female rats, divided into five groups, underwent 14 days of treatment. Bone biomechanical properties were assessed via finite element analysis based on micro - CT.Camargos et al. found that high-frequency vibration increased cortical bone width in ovariectomized rats but failed to improve biomechanical properties, whereas alendronate prevented trabecular bone degradation and enhanced bone hardness and strength. When combined, these interventions synergistically increased cortical thickness and further improved therapeutic outcomes (33).Stuenner et al. explored using estrogen (E) and raloxifene (R) with 70 Hz whole-body vibration (WBV) twice daily for six weeks to enhance bone healing in 84 ovariectomized rats. Results showed E and R improved osteopenic bone structure, as did WBV alone, though WBV rarely reached significance. Combination treatments significantly boosted stiffness, endosteal bone, and trabecular density. Trap expression was also reduced. The additive effects suggest WBV with E or R may aid osteopenic bone healing (34).

Butezloff et al. examined vibration therapy’s impact on fractured femur bone callus and intact femur bone quality in ovariectomized rats. Bone and callus quality were assessed by densitometry, 3D microstructure, and mechanical tests. Ovariectomized rats showed significant bone mass loss and microarchitecture impairment. Vibration therapy improved bone and fracture callus parameters in osteoporotic bone, enhancing bone quality and fracture callus in ovariectomized rats (35).

Qing et al. tested 16 - week low - magnitude, high - frequency vibration (LMHFV) on osteoporotic and healthy rats. It found that at week 8, LMHFV improved OVX - induced trabecular bone deterioration, but this effect didn’t last. Osteoblasts from osteoporotic rat bone showed short - term positive gene expression changes from LMHFV, but no lasting benefits (36). A randomized controlled trial by Chen et al. indicated that combining vibration therapy with bisphosphonate treatment for 3 months in ovariectomized rats significantly enhanced bisphosphonate efficacy, improved bone metabolism, and exhibited synergistic effects in preventing osteoporosis and optimizing trabecular structure, confirming vibration therapy’s role in augmenting bisphosphonate anti-osteoporotic effects (37). However, some studies argue that while mechanical vibration promotes cortical bone synthesis and repairs damaged bone tissue, it exerts no significant impact on trabecular structure. Microstructural analysis showed that combining alendronate with mechanical vibration did not further enhance therapeutic outcomes (38, 39).

Although preclinical studies confirm vibration therapy’s osteogenic potential (**Table 1**), clinical trial results remain inconsistent (**Table 2**). Most research supports its efficacy in improving BMD. Lai et al. found that high-frequency, high-intensity whole-body vibration applied to postmenopausal women for 6 months increased lumbar spine BMD by 2.032% from baseline, significantly outperforming controls as measured by dual-energy X-ray absorptiometry (40). Jepsen et al. explored teriparatide combined with vibration therapy, revealing a 2.95% greater lumbar BMD increase in the combination group compared to teriparatide alone, though no statistical differences were observed in hip BMD or bone microstructure parameters (41). These findings suggest vibration therapy’s conditional efficacy in enhancing BMD.

**Table 1:** Basic Experimental studies on vibration therapy for osteoporosis.

References	Research subjects	Therapy parameters (duration, timing or frequency)	Observation indicators	Results
(32)	Ovariectomized rats	A frequency of 30–35 Hz and an acceleration of 0.3 g twice a day for 20 min with a 5 min rest at the mid‐point (10 mins on, 5 mins off, 10 mins on) for 5 days/week for 12 weeks	Concentrations of osteopontin The amount of bone turnover	Effective
(33)	Ovariectomized rats	A protocol that consisted of 10 consecutive frequency steps (130, 135, 140, 145, 150, 130, 135, 140, 145 and 150 Hz), each of these applied for 1 minute at an acceleration of 0.3g	Bone stiffness and bone strength	Effective
(34)	Estrogen-deficient rats	A frequency of 70 Hz with an amplitude of 0.4 mm,for 15 min 2 times per day for 37 days	Bone stiffness, endosteal bone and trabecular density	Effective
(35)	Ovariectomized rats	Whole-body vibration therapy, with peak-to-peak vertical displacement of 1.0 mm at a frequency of 60.0 Hz, 3 days per week for 20 minutes per session for 14 or 28 days	Bone quality and the quality of the fracture bone	Effective
(36)	Ovariectomized rats	A 0.3 g, 30 Hz LMHFV regimen, 20 min/day for 16 weeks	Bone mineral densities, bone mechanical properties and cellular responses	Effective
(37)	Ovariectomized rats	0.3 g at 45–55 Hz for continuous 20 min/day, 5 day/week, for 3 months	The bone morphology and density parameters	Effective
(38)	Ovariectomized rats	A protocol that consisted of 10 consecutive frequency steps (130, 135, 140, 145, 150, 130, 135, 140, 145, and 150 Hz), each of these applied for 1 min at an acceleration of 0.3g, 10 min/day	Trabecular bone micro-architecture	Invalid
(39)	Ovariectomized rats	A protocol that consisted of 10 consecutive frequency steps (130, 135, 140, 145, 150, 130, 135, 140, 145 and 150 Hz), each of these applied for 1 minute at an acceleration of 0.3g	Trabecular patter factor (Tb.Pf), trabecular separation (Tb.Sp), andstructure model index	Invalid

**Table 2:** Clinical trials of vibration therapy for osteoporosis.

References	Research subjects	Therapy parameters (duration, timing or frequency)	Observation indicators	Results
(40)	Postmenopausal women	A frequency of 30 Hz and a magnitude of 3.2 g for 5 minutes each time	Bone mineral density of the lumbar	Effective
(41)	Postmenopausal osteoporotic women	A frequency of 30 Hz and amplitude of 1 mm (low displacement) and peak acceleration of 35.53 ms^−2^ root-mean-square (3.6 g)	Bone mineral density and bone microarchitecture, bone turnover markers, and sclerostin measurements	Effective
(42)	Postmenopausal women	Short-duration (two 10-minute treatments/day), low-magnitude (2.0 m/s^2^ peak to peak), 30-Hz vertical accelerations (vibration)	Bone mineral density at the spine, hip, and distal radius	Effective
(43)	Postmenopausal women	the amplitude (low, 1.7 mm; high, 2.5 mm) and/or the frequency (35–40 Hz) of the vibration, the duration of the WBV program was a maximum of 30 minutes	Isometric and dynamic musclestrength ,bone mineral density of the hip, serum markers of bone turnover	Effective
(44)	distal radius density in adults	Whole body vibrations on the vibration platform (35 Hz, 0.25 g) once a day, for 15 minutes per session over a period of 4 weeks	The bone mineral density of distal radius	Effective
(45)	Postmenopausal women	The influence of twice-weekly low-intensity whole body vibration (15 mins, 30 Hz, 0.3 g) or higher intensity whole body vibration (2 × 3 mins, 12.5 Hz, 1 g)	Bone loss at the hip and spine , lower limb muscle function	Effective
(46)	Elderly people	The frequency of vibration was 40 Hz and the amplitude was 2 mm (peak to peak),3 times per week for 11 weeks, 10 repetitions of 45 s with a rest period of 60 s between each repetition	The bone mineral content and bone mineral density parameters	Invalid

A 1 - year trial of 70 postmenopausal women found brief low - intensity (0.2g, 30Hz) vibration can curb spinal and femoral bone loss, with effects improving with compliance, especially in lighter women. Animal models show low - intensity vibration enhances trabecular structure and cancellous bone strength. Results showed compliance significantly impacts efficacy, with high - compliance groups seeing a 2.17% and 1.5% improvement in femoral neck and spinal BMD. In women under 65kg with high compliance, spinal BMD improved by 3.35% (42). Another study showed a 0.93% increase in hip BMD after 6 months of vibration therapy in postmenopausal women (43). Tan et al.assessed the effect of 4 - week whole - body vibration (35Hz, 0.25g) on distal radius bone mineral density (rBMD) in 114 adults with osteoporosis or osteopenia. After treatment, average rBMD increased by 1.79% (P<0.05), with increases of 1.77% and 1.80% in men and women, respectively (both P<0.05) (44). Beck et al. observed the effect of whole-body vibration on hip fracture risk factors in postmenopausal women. 47 women completed the trial. The control group showed significant bone density loss at the hip and spine, while the vibration group did not. The vibration group also had significant improvements in wall squats and chair rises. Although no significant between-group differences were found, whole-body vibration may reduce bone loss and improve lower limb muscle function, thus lowering the risk of falls and hip fractures (45). These results highlight vibration therapy’s potential clinical value in improving BMD and reducing fracture risk.

However, some studies question its efficacy. Gómez-Cabello et al. observed no significant changes in femoral neck, hip, or lumbar spine BMD or bone mass after 11 weeks of vibration therapy (40 Hz, 2 mm amplitude, 20 minutes/session, 3 sessions/week) in 24 elderly participants (46). Luo et al. further argued that vibration therapy lacks systemic therapeutic effects on BMD or bone turnover markers in postmenopausal osteoporotic patients. They attributed this to vibration signal attenuation during transmission and site-specific bone responses, potentially influenced by musculoskeletal nonlinearity (e.g., joint angles, soft tissue distribution) and posture-dependent vibration transmission rates (e.g., bent knees vs. straight legs) (47).

Discrepancies in clinical outcomes may also stem from variations in vibration parameters (frequency, amplitude) and equipment across studies.

## 5 Summary

Vibration therapy, as a non-pharmacological and non-invasive therapeutic approach, achieves multiple regulatory effects on bone metabolism through low-load mechanical stimulation: promoting osteoblast differentiation and proliferation, inhibiting osteoclast activity, reducing bone turnover rate, increasing bone density, and optimizing bone microstructure, ultimately reversing the pathological progression of osteoporosis. Compared to traditional pharmacological and exercise therapies, this therapy offers significant clinical advantages: it avoids the economic burden of long-term medication and the risk of adverse drug reactions, its effectiveness is not constrained by the technical skill level of the practitioner, it requires short single-treatment sessions (typically 5–20 minutes), is easy to operate, and does not require patients to actively participate in high-intensity exercise. These features significantly improve treatment compliance, making it particularly suitable for patients with mobility impairments, frailty, or disability.

Although existing research has confirmed the efficacy of vibration therapy for osteoporosis, key issues such as determining the optimal vibration parameters (frequency/amplitude/acceleration combinations) and the impact of device parameter heterogeneity on therapeutic outcomes remain unresolved, and there is currently a lack of authoritative clinical guidelines to guide practice. FremTherefore, establishing an evidence-based system of personalized treatment parameters and clarifying the biological equivalence of treatment doses across different devices will be the core research directions for advancing the standardization of this therapy in clinical practice.
